# Efficient Excitation and Tuning of Multi-Fano Resonances with High Q-Factor in All-Dielectric Metasurfaces

**DOI:** 10.3390/nano12132292

**Published:** 2022-07-04

**Authors:** Yunyan Wang, Chen Zhou, Yiping Huo, Pengfei Cui, Meina Song, Tong Liu, Chen Zhao, Zuxiong Liao, Zhongyue Zhang, You Xie

**Affiliations:** 1 Xi’an Key Laboratory of Optical Information Manipulation and Augmentation, School of Physics and Information Technology, Shaanxi Normal University, Xi’an 710062, China; yyywang@snnu.edu.cn (Y.W.); 18291927192@163.com (C.Z.); pfcui@snnu.edu.cn (P.C.); 202017027@snnu.edu.cn (M.S.); lt20211965@snnu.edu.cn (T.L.); zhaochen1999@snnu.edu.cn (C.Z.); zxliao@snnu.edu.cn (Z.L.); zyzhang@snnu.edu.cn (Z.Z.); 2 College of Science, Xi’an University of Science and Technology, Xi’an 710054, China; xieyou@xust.edu.cn

**Keywords:** Fano resonance, anapole resonance mode, toroidal dipole (TD) mode, quality factor (Q-factor)

## Abstract

Exciting Fano resonance can improve the quality factor (Q-factor) and enhance the light energy utilization rate of optical devices. However, due to the large inherent loss of metals and the limitation of phase matching, traditional optical devices based on surface plasmon resonance cannot obtain a larger Q-factor. In this study, a silicon square-hole nano disk (SHND) array device is proposed and studied numerically. The results show that, by breaking the symmetry of the SHND structure and transforming an ideal bound state in the continuum (BIC) with an infinite Q-factor into a quasi-BIC with a finite Q-factor, three Fano resonances can be realized. The calculation results also show that the three Fano resonances with narrow linewidth can produce significant local electric and magnetic field enhancements: the highest Q-factor value reaches 35,837, and the modulation depth of those Fano resonances can reach almost 100%. Considering these properties, the SHND structure realizes multi-Fano resonances with a high Q-factor, narrow line width, large modulation depth and high near-field enhancement, which could provide a new method for applications such as multi-wavelength communications, lasing, and nonlinear optical devices.

## 1. Introduction

The quality factor (Q-factor) can represent the energy limiting efficiency of optical interaction between the external environment and a material. High Q-factor resonators have an important role in various fields of optical applications, such as nonlinear optics [1,2,3], optics sensors [4], lasers [5], and optical switches [6]. Fano resonance, produced by the destructive interference between discrete and continuous states, can form a typical sharp asymmetrical line shape and is an effective method used to achieve a high Q-factor in metasurfaces [7,8]. In the past few years, researchers have discovered that metal metasurfaces based on plasmonic resonance can generate Fano resonance [9,10]. However, a clear shortcoming is that the inherent ohmic loss of a metal metasurface is very large, which reduces the utilization rate of light energy [11]. Meanwhile, Fano resonances formed in the metal metasurface are mainly electric resonances, such as electric dipole, electric quadrupole, and high-order electrode modes, which will also cause serious radiation loss and render Q-factor enhancement difficult [12]. For both cases, because ohmic loss is an inherent property of a metal metasurface and is difficult to change, there is a need to design a special metal metasurface structure, such as the split ring structure [13,14], to excite the magnetic resonance modes and reduce energy loss, which can increase the Q-factor. Unfortunately, this method can limit the freedom of optical device design and severely limit practical applications.

Several studies have shown that a high-refractive-index all-dielectric metasurface [15,16] can significantly reduce ohmic and radiation losses, which can achieve a high Q-factor Fano resonance not matchable by a metal metasurface based on plasmon resonance. Compared to a metamaterial based on plasmonic resonance, where resonances are often dominated by electric resonance modes, all-dielectric metasurfaces can support a series of BIC and Mie resonances. Bound states in the continuum (BIC) represents an ideal physical system, which can also be referred to as capture mode. The energy is confined in the resonant cavity, showing zero radiation loss, and has the characteristic of an infinite Q-factor. By breaking the symmetry of the all-dielectric metasurfaces, a channel for energy leakage into free space is provided and BIC can be converted into quasi-BIC with a finite Q-factor, which is called the leakage mode. It is an effective method to obtain a high Q-factor in all-dielectric metasurfaces [17,18,19,20]. Mie resonance means that when electromagnetic waves are irradiated on dielectric particles, they will interact with each other to realize scattering in response to electric or magnetic fields [21,22]. This can not only generate electric resonance modes but also generate magnetic and toroidal resonance modes [23,24], thereby significantly reducing radiation loss. Currently, electric dipole (ED), magnetic dipole (MD) and toroidal dipole (TD) resonances are considered to represent the three types of fundamental modes of electromagnetics. ED resonance is generated from the separation of negative and positive charges; MD resonance is induced by the closed circulation of electric current; and TD resonance can be produced by a pair of adjacent current loops with opposite MD moments, as proposed by Zel’dovich in 1957 [25] and first experimentally observed in 2010 [26]. In an all-dielectric metasurface, the fundamental resonance is a series of magnetic resonance modes, which are useful for the confinement of the incident field within the metamaterial, leading to near-field enhancement inside the dielectric devices so that magnetic Fano resonance can be easily induced [27,28]. When the ED and TD resonances overlap, scattering the amplitude and antiphase, the non-radiating anapole mode can be generated [29,30]. Owing to the non-radiating character and efficient energy confinement of the anapole mode, a high Q-factor resonance can be achieved, which has important application in nano-lasers [31,32] and enhances nonlinear effects [33,34].

As the wide application of multiplexing methods in various devices has developed, the research focus on optical Fano resonance has also expanded from single Fano resonance to multi-Fano resonance [35,36]. Multi-Fano resonance can be used efficiently in a multi-channel biosensor and multi-band slow-light device. As recent studies have shown, multi-Fano resonance is useful in enhancing multi-band multi-harmonic generation, such as second and third harmonic generation [37], where different Fano resonances match different fundamental wavelengths and different harmonic wavelengths.

In this article, an all-dielectric silicon square-hole nano disk (SHND) array metasurface was designed and evaluated. Similar structures have been proposed previously. For example, Algori et al designed two metasurfaces based on hollow nanostructures with Q-factors of 2.5 × 10^6^ and 1.71 × 10^6^, but only one Fano resonance was excited [38,39], while Jeong et al. designed a dielectric metasurface structure in which two Fano resonances were excited with a Q-factor of 728 [40]. In the present investigation, by adjusting the inner square along the *x*-axis and breaking the symmetry of the all-dielectric metasurface, the BIC mode with an infinite Q-factor was transformed into a quasi-BIC mode with finite size, which induced triple Fano resonance. The influence of the extinction coefficient on Q-factor was investigated. Then, applying multilevel decomposition theory, it was found that the anapole, toroidal, magnetic quadrupole, and MD resonance modes were excited simultaneously. All the three Fano resonances were based on magnetic resonance and showed a high Q-factor because of weak radiative and non-radiative decay. The maximum Q-factor was able to reach 35,837 in magnitude and the modulation depths of all the Fano resonances were nearly 100%. In addition, the electromagnetic near-field enhancements could be confined inside the metasurface and were greatly enlarged by breaking the symmetry of the SHND nanostructure, which provided an effective method to modulate the localized field. Therefore, the SHND structure could operate in multi-wavelength communications, lasing, and nonlinear optical devices.

## 2. Materials and Methods

**Figure 1:**
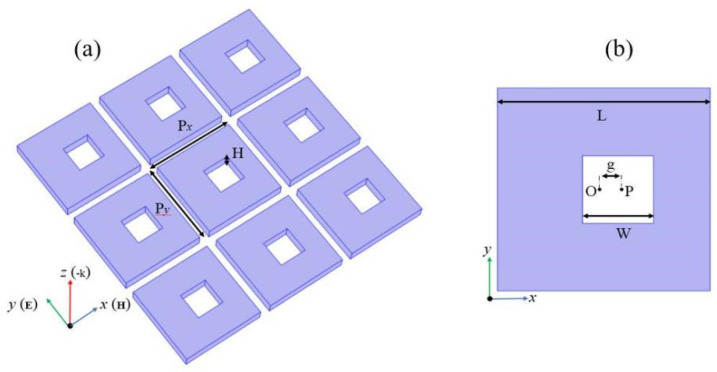
(**a**) Schematic of the SHND nanostructure array and the incident light polarization configuration; (**b**) Top view and geometric parameters of a unit cell of the SHND.

The SHND nanostructure array, shown in Figure 1, was placed on a glass substrate with a refractive index of 1.5 and completely submerged in water with a refractive index of 1.33. The thickness of the entire structure is H, the side length of the outer square with center O is L, the side length of the inner square hole with center P is W, and the distance between points O and P is defined as an asymmetry parameter g. The material that constituted the SHND structure was amorphous silicon, and the optical parameters were obtained from experimental data [41]. The initial parameters of the SHND structure were L = 600 nm, W = 200 nm, H = 100 nm, g = 0 nm, and the periodic parameters of the unit cell were Px = Py = 680 nm. The incident plane wave enters the SHND structure along the *z*-axis, whereas the polarization of the electric field follows the positive direction of the *y*-axis. All the simulation results were obtained using COMSOL Multiphysics 5.6 software, which is based on the finite element method. A three-dimensional SHND model was built in the software, and the structure was subdivided by a free triangular mesh. The degrees of freedom were 14,238 and the periodic boundary conditions were adopted as the boundary conditions. The SHND nanostructure can be prepared using a standard nano-process, as follows: firstly, deposit a thin silicon film on a silicon dioxide substrate using a low-pressure physical vapor deposition (LPCVD) method; secondly, etch rectangular holes using electron beam lithography (EBL), or a reactive ion-etching method; and finally, remove the photoresist and rinse the nanostructure with deionized water.

## 3. Theory

The investigation of the electromagnetic properties of the dielectric nanostructures is based on the decomposition of multipole moments in a Cartesian coordinate system. A general expression for multipoles is as follows:

Electric dipole moment:

(1) $$p=\frac{1}{j\omega }\int_{V}J(r)dr$$

Magnetic dipole moment:

(2) $$m=\frac{1}{2\upsilon_{b}}\int_{V}\left[r\times J(r)\right]dr$$

Toroidal dipole moment:

(3) $$t=\frac{1}{10\upsilon_{b}}\int_{V}\left[\left(r\cdot J(r))r-2r^{2}J(r\right)\right]dr$$

Electric quadrupole moment:

(4) $$Q_{\alpha \beta }^{e}=\frac{1}{j2\omega }\int_{V}[r_{\alpha }J_{\beta }+r_{\beta }J_{\alpha }-\frac{2}{3}\delta_{\alpha \beta }(r\cdot J(r))]dr$$

Magnetic quadrupole moment:

(5) $$Q_{\alpha \beta }^{m}=\frac{1}{3\upsilon_{b}}\int_{V}\{[r\times J(r){]}_{\alpha }r_{\beta }+{\left[r\times J(r)\right]}_{\beta }r_{\alpha }]\}dr$$

where ***j*** denotes the current density, *υ_b_* the speed of light in the medium, **r** is the radial vector, and the subscripts for the electric and magnetic quadrupoles are *α*, *β* = *x*, *y*, *z*. When the scatter wavelength is smaller than the incident wavelength, the higher-order terms (e.g., octupole) can be neglected.

The radiation powers for different multipole moments are given as follows:

(6) $$I_{P}=\frac{2\omega^{4}}{3\upsilon_{b}^{3}}{\left|p\right|}^{2}$$

(7) $$I_{t}=\frac{2\omega^{6}}{3\upsilon_{b}^{5}}{\left|t\right|}^{2}$$

(8) $$I_{m}=\frac{2\omega^{4}}{3\upsilon_{b}^{3}}{\left|m\right|}^{2}$$

(9) $$I_{Qe}=\frac{\omega^{6}}{5\upsilon_{b}^{5}}{\left|Q_{\alpha \beta }^{e}\right|}^{2}$$

(10) $$I_{Qm}=\frac{\omega^{6}}{40\upsilon_{b}^{5}}{\left|Q_{\alpha \beta }^{m}\right|}^{2}$$

This multipole decomposition method allows for the identification of the contributions stemming from TD moments and hence identification of the conditions for the anapole mode excitation [42,43,44].

## 4. Results and Discussion

### 4.1. Excitation of Fano Resonance in the SHND Structure and Influence Factors of Q-Factor

**Figure 2:**
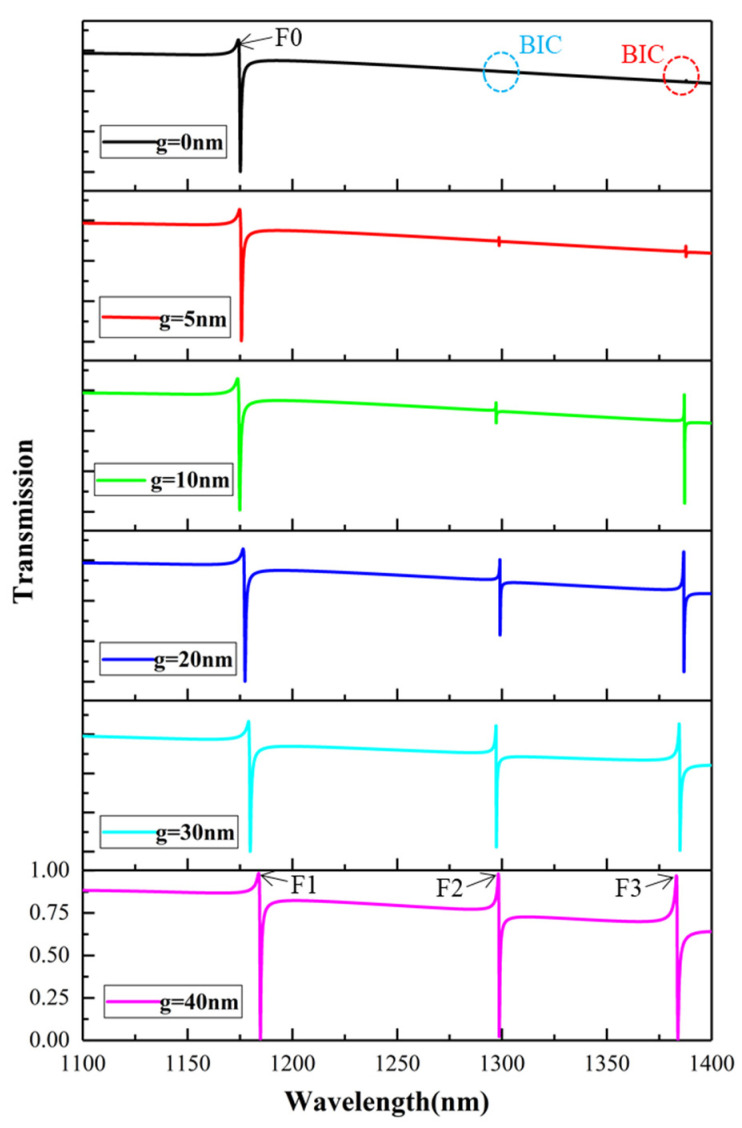
Transmission spectra of the SHND nanostructure with different asymmetry parameters.

**Figure 3:**
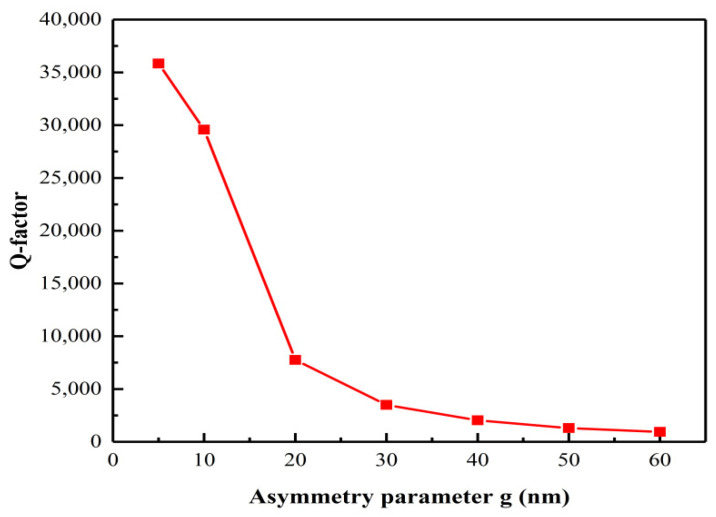
Q-factor of F3 with different asymmetric parameters.

Figure 2 shows the transmission spectra of the proposed metasurface at different asymmetry parameters. When g = 0 nm, the square hole is located at the center of the entire structure. In this case, points O and P coincide and the SHND nanostructure is completely symmetrical. There is only one asymmetric Fano resonance in the transmission spectrum. As shown in Figure 2, when g = 0 nm, there is no transmission peak except F0, which means that the Q-factor tends to infinity, showing a BIC mode. When the inner square hole moves horizontally to the right, the symmetry of the SHND nanostructures is broken; there are two obvious transmission dips near λ = 1294.9 nm and λ = 1389.5 nm, which show obvious Fano characteristics. This means that the BIC state becomes unstable with increase in the asymmetric parameter, and the breaking of symmetry provides a zero-order radiation channel for the metasurface, which leads to leakage of energy and the BIC mode is transformed into a quasi-BIC mode. The larger the asymmetry parameter, the greater the radiation loss energy and the smaller the Q-factor. When g = 40 nm, three Fano resonances appear in the transmission spectrum, which are denoted by F1, F2, and F3. Figure 2 shows that their resonance peaks are at 1182.2 nm, 1294.9 nm, and 1389.5 nm, respectively. The Q-factor is defined by Q = $\;\omega_{0}$/2γ  [45,46], where $\omega_{0}$ is the resonance frequency and γ is the damping loss. Figure 3 shows the variation in the Q-factor of F3 with different asymmetric parameters. When the asymmetry factor is 5 nm, the Q-factor can reach 35,837. With increase in the asymmetry parameter g, the Q-factor decreases rapidly.

**Figure 4:**
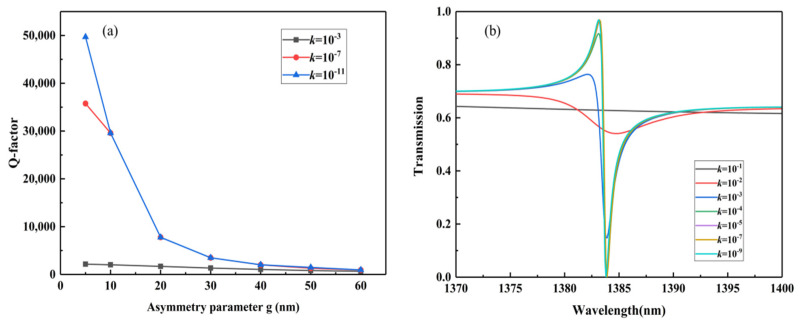
(**a**) Q-factor of F3 at *k* = 10^−3^, 10^−7^ and 10^−11^ with different asymmetric parameters. (**b**) Transmission spectra of F3 with different extinction coefficients *k* at g = 40 nm.

In practical applications, errors in the Q-factor may be caused by factors such as technology and the environment. For example, the actual all-dielectric metasurface is uneven, which will lead to an increase in transmission loss and decrease in the Q-factor. As the substrate in our simulation is transparent glass, loss of silicon is the main consideration. By adding an extinction coefficient *k* (i.e., the imaginary part of the refractive index of silicon) [47], the results can include the absorption and scattering losses caused by surface roughness in the real manufacturing process. As shown in Figure 4a, when *k* = 10^−11^ or 10^−7^, the Q-factors decrease as g increases; when *k* = 10^−3^, the Q-factor maintains nearly the same value. Figure 4b shows the F3 resonance curves with different *k* values of g = 40 nm. It can be seen that when *k* = 10^−1^, there is no transmission peak in the F3 wavelength range; when *k* is less than 10^−1^, the transmission peak gradually appears in the F3 wavelength range, and the Q-factor increases accordingly. This is because decrease in the *k* value leads to decrease in loss.

### 4.2. Multipole Decomposition of Fano and Analysis of Its Resonance Mode

To gain insight into the optical properties of the designed multi-Fano resonance device and to identify the contribution of different multipole resonance modes to the Fano resonances, the electric field and magnetic field distributions were calculated.

**Figure 5:**
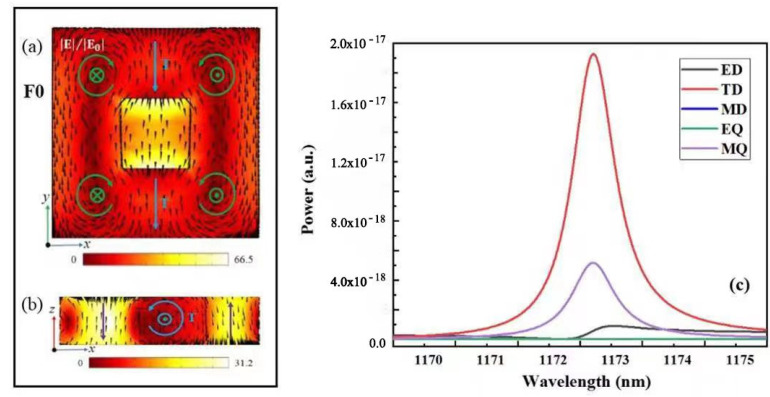
When g = 40 nm, the SHND nanostructure is symmetric. (**a**) Electric field enhancement and electric field vector distribution of F0 in the x–y plane. (**b**) Magnetic field enhancement and magnetic field vector distribution of F0 in the x–z plane. (**c**) Radiant energy of each multipole resonance mode at F0.

To understand the specific characteristics of the Fano resonance with g = 0 nm, the electric and magnetic field distributions at the resonance peak of 1172.6 nm are shown in Figure 5a,b. From the electric field distribution in Figure 5a, it can be observed that the SHND structure generates four magnetic loop currents on the x–y plane simultaneously. The MD moments of the two magnetic loop currents on the left are along the negative direction of the *z*-axis, whereas the MD moments of the two magnetic current loops on the right are along the positive direction of the *z*-axis. The TD resonance is generated, and its direction points to the negative direction of the *y*-axis, which is shown by the blue arrows in Figure 5a,b. This result coincides with the radiant energy distribution of each multipole resonance mode of the SHND structure with g = 0 nm, as shown in Figure 5c, indicating that the TD mode is dominant at F0.

**Figure 6:**
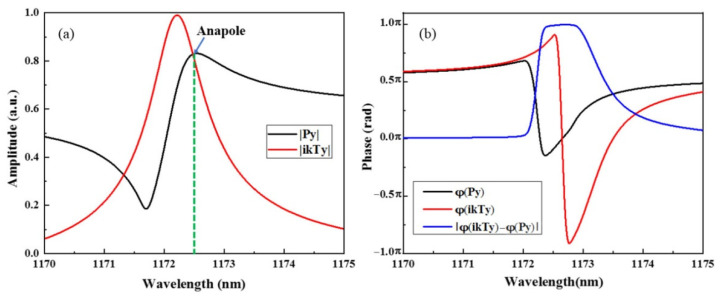
(**a**) Amplitude of the Cartesian electric dipole moment |Py| and toroidal dipole moment |ikTy| of F0 when g = 0 nm. (**b**) Phases, with the differences of Py and ikTy.

However, further analysis shows that the amplitudes of the current Cartesian ED moment |Py| and the TD moment |ikTy| are nearly equal at 1172.6 nm, as shown in Figure 6a. Meanwhile, the phase difference of the ED and TD moments |φ(ikTy) − φ(Py)| is approximately equal to π, as shown in Figure 6b. The far-field radiation of the ED and TD resonances interfere destructively with each other; thus, the subradiant resonance anapole mode can be induced. In addition to the destructive interference between ED and TD resonances, the incident field can be efficiently trapped into the unit cell of the SHND metasurface, which plays an important role in the shaping of the resonance line type.

**Figure 7:**
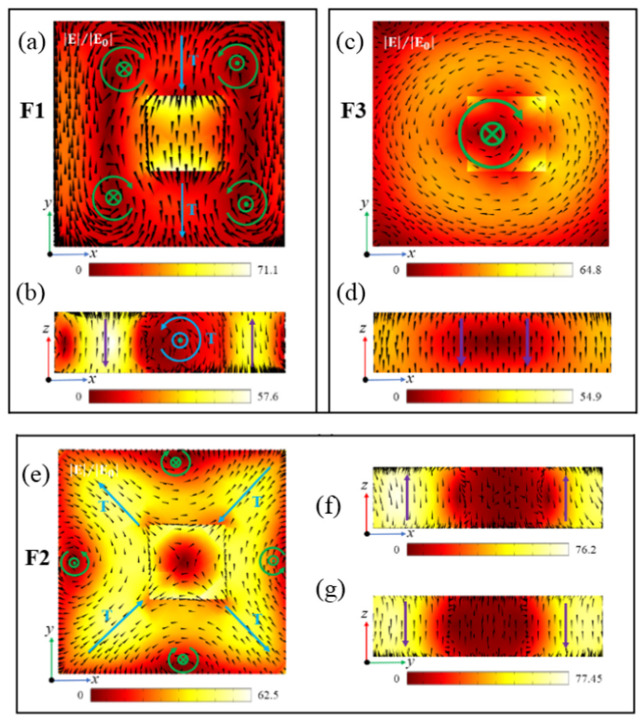
When g = 40 nm, the symmetry of the SHND nanostructure is broken. (**a**,**c**,**e**) show the electric field enhancement and electric field vector distribution of F1, F2, and F3 in the *x*–*y* plane. (**b**,**d**,**f**) show the magnetic field enhancement and magnetic field vector distribution of F1, F2, and F3 in the *x*–*z* plane. (**g**) shows the magnetic field enhancement and magnetic field vector distribution of F2 in the *y*–*z* plane.

**Figure 8:**
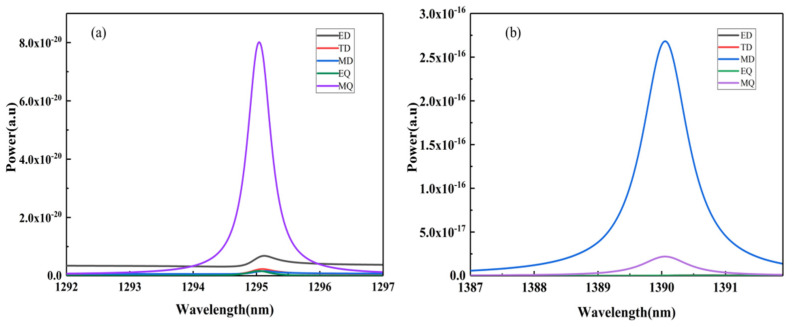
Radiant energy of each multipole resonance mode at (**a**) F2, and (**b**) F3.

Figure 7 shows the electromagnetic field distributions of the three Fano resonances of the SHND structure with g = 40 nm. Figure 7a,b show that the electromagnetic field enhancement of F1 is greater than that of F0 and the electromagnetic field distribution of F1 changes slightly. Figure 7e shows the electric field distribution of F2 at 1294.9 nm in the *x*–*y* plane; there are also four MD resonances generated by the four magnetic loop currents in the middle of the four outer edges of the square cavity. From the combination of the magnetic field distribution in the *x*–*z* and *y*–*z* planes shown in Figure 7f,g, it can be inferred that two of the four magnetic loop currents in Figure 7e can be combined with each other to form TD resonance modes. Thereafter, these TD resonance modes are formed in the diagonal direction of the SHND structure, and the resonance moment directions of the two TD resonance modes on the same diagonal line are opposite. This resonance mode can be regarded as magnetic quadrupole (MQ) resonance. The multipole decomposition of F2 is shown in Figure 8a, where the energy of the magnetic quadrupole (MQ) moment is the largest, indicating that the generation of FR2 is mainly due to MQ resonance. Figure 7c,d show the electric field distribution and magnetic field distribution of F3 in the *x*–*y* plane and *x*–*z* plane, respectively. It can be seen from the figure that the electric field in the *x*–*y* plane forms a ring current, and that there is a negative direction of the magnetic field along the *z*-axis. The multipole decomposition of F3 is shown in Figure 8b. It can be seen that the energy of the MD moment is the largest, so F3 is dominated by MD resonance, and the MD moment is along the negative direction of the *z*-axis.

By continuing to analyze the electromagnetic field enhancement features of F1, F2, and F3, it can be determined that the electromagnetic field enhancements of the three Fano resonances are nearly three times stronger than the electromagnetic field enhancement of the golden cross-shaped dimer structure [48]. In particular, the maximum electric field enhancements of F1 and F2 are found to be 71.1- and 77.45-fold, respectively; therefore, the SHND nanostructure can be applied in areas where significant near-field enhancement is required.

In addition, the modulation depth [49,50] is an effective parameter of Fano resonance, which can be used to describe the intensity range of Fano resonance. The modulation depth is usually defined as Δ*T* = *T*_peak_ − *T*_dip_, where *T*_peak_ and *T*_dip_ are the intensities of the resonance peak and dip, respectively. According to this definition, the modulation depths of F0, F1, F2, and F3 of the SHND structure at g = 0 nm and g = 40 nm are all reached at nearly 100%, which provides a technique for obtaining equipment with high modulation depth.

### 4.3. Influence of Geometric Parameters on F1, F2, and F3 of the SHND Structure

**Figure 9:**
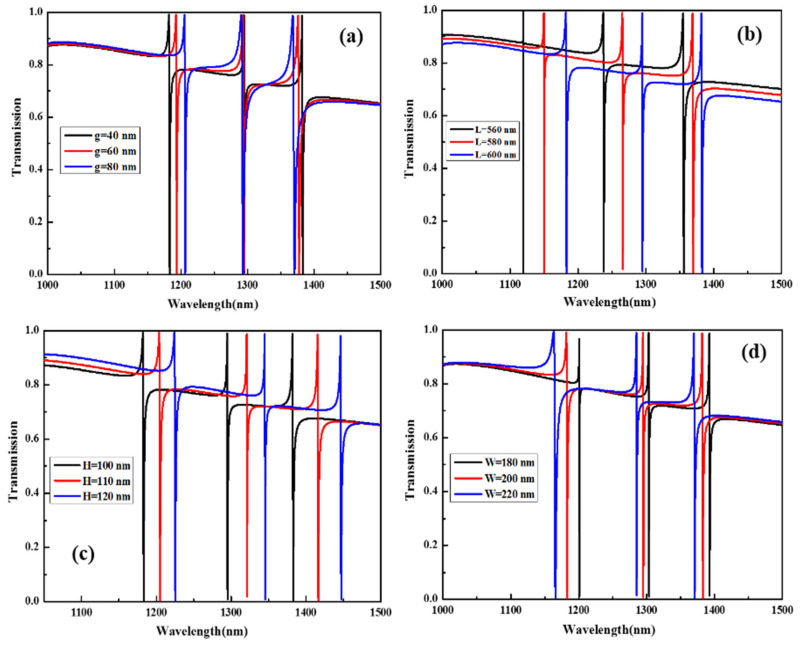
Transmission spectra of multi-Fano resonance of the SHND structure with different (**a**) asymmetry parameter g, (**b**) structure height H, (**c**) outer edge length L, and (**d**) inner cavity width W.

To study the dependence of the transmission spectra characteristics on the different geometric parameters of the SHND structure, the corresponding transmission spectra characteristics to variable parameters when g = 40 nm were calculated and are shown in Figure 9.

As the asymmetry parameter *g* value increases, it can be observed from Figure 9a that the transmission spectra of F1 undergoes a significant red shift and F3 exhibits a significant blue shift. Compared with F1 and F3, the position of F2 can be considered as not moving, which shows that F2 is not sensitive to changes in *g*. In addition, with increase in *g*, the resonance modes F1, F2, and F3 are all widened; in Figure 9b, with decrease in the outer length L, the three Fano resonances are blue-shifted. The reason is that with decrease in L, the surface area of SHND also decreases, resulting in a decrease in the effective refractive index. In Figure 9c, with increase in the SHND structure height H, the three Fano resonances show a significant simultaneous red-shift, because the increase in H leads to an increase in the effective refractive index of the SHND surface. At the same time, an increase in H has little effect on the resonant line width and Q-factor. It can also be observed from Figure 9d that, with increase in the width of the inner cavity W, the three Fano resonances also exhibit a clear blue-shift. The reason is that increase in W leads to a decrease in the SHND surface area, which leads to a decrease in the effective refractive index of the surface.

By comprehensively comparing and analyzing the three Fano resonance characteristics in the four cases, we can confirm that F1 is very sensitive to changes in the outer edge length L, the structural height H, and the internal cavity width W. F2 is insensitive to changes in the asymmetry parameter g, and sensitive to changes in the width of the inner cavity W, the outer length L, and the structure height H. F3 is most sensitive to changes in the structure height H. The above results show that, in practical applications, the multi-Fano resonance of the SHND metasurface can be adjusted flexibly according to the specific conditions. In addition, when g, L, H, and W are all changed in different ways, the modulation depths of the three Fano resonances F1, F2, and F3 generated by the SHND structure are all practically 100% and their FWHMs are very narrow. This result supports the design of optical devices with multiple Fano resonance, a high Q-factor, and high modulation depth.

## 5. Conclusions

We have demonstrated that, by breaking the symmetry of all-dielectric SHND metasurfaces and converting the BIC mode into a quasi-BIC mode, multi-Fano resonance with a high Q-factor and nearly 100% modulation depth can be obtained. The influence of the extinction coefficient *k* on the Q-factor was also examined. Due to the formation of subradiation hybrid resonance modes, such as anapole and TD resonance modes, the light energy efficiency of the SHND metasurface interacting with incident light is increased, resulting in Fano resonances producing extremely narrow FHMWs and higher Q-factors. Moreover, the maximum Q-factor was found to reach 35,837 in magnitude. In addition, by changing the geometric parameters of the SHND, a larger Q-factor and a wide range of adjustments of the Fano resonance positions can be obtained. Therefore, this type of multi-Fano resonance with a high Q-factor and non-localized characteristics can enhance adaptability, enabling the SHND structure to be applied to optoelectronics and nonlinear optical devices in the visible light range, achieving higher-efficiency large-scale optoelectronic integration.

## Data Availability

The data presented in this study are available on request from the corresponding author.
